# Behavioral responses to odors from other species: introducing a complementary model of allelochemics involving vertebrates

**DOI:** 10.3389/fnins.2015.00226

**Published:** 2015-06-25

**Authors:** Birte L. Nielsen, Olivier Rampin, Nicolas Meunier, Vincent Bombail

**Affiliations:** ^1^Department of Animal Physiology and Livestock Systems, INRA, UR1197 NeuroBiologie de l'OlfactionJouy-en-Josas, France; ^2^Department of Biology, Université de Versailles Saint-Quentin-en-YvelinesVersailles, France

**Keywords:** olfaction, interspecific interactions, semiochemicals, allomones, kairomones, odor valence, innateness, learning

## Abstract

It has long been known that the behavior of an animal can be affected by odors from another species. Such interspecific effects of odorous compounds (allelochemics) are usually characterized according to who benefits (emitter, receiver, or both) and the odors categorized accordingly (allomones, kairomones, and synomones, respectively), which has its origin in the definition of pheromones, i.e., intraspecific communication via volatile compounds. When considering vertebrates, however, interspecific odor-based effects exist which do not fit well in this paradigm. Three aspects in particular do not encompass all interspecific semiochemical effects: one relates to the innateness of the behavioral response, another to the origin of the odor, and the third to the intent of the message. In this review we focus on vertebrates, and present examples of behavioral responses of animals to odors from other species with specific reference to these three aspects. Searching for a more useful classification of allelochemical effects we examine the relationship between the valence of odors (attractive through to aversive), and the relative contributions of learned and unconditioned (innate) behavioral responses to odors from other species. We propose that these two factors (odor valence and learning) may offer an alternative way to describe the nature of interspecific olfactory effects involving vertebrates compared to the current focus on who benefits.

## Introduction

Odors can influence the behavior of animals. Behind this simple statement is a hidden world of complex links and interactions, and a large body of scientific studies dealing with various aspects from chemo-sensitivity and implicated brain regions, to evolutionary pathways and functionality. One area of olfactory behavior research is the study of odor-based effects between organisms. These odors are referred to as semiochemicals (Law and Regnier, 1971; Regnier, 1971), and consist of two major groups: pheromones [Karlson and Lüscher, 1959a; originally named ectohormones by Bethe (1932)] for intraspecific interactions, and allelochemics (Whittaker, 1970) for interactions between organisms of different species. Allelochemics are thus odors by which members of one species affect the growth, health, or behavior of members of another species (Whittaker and Feeny, 1971).

Allelochemics were initially divided into two groups, consisting of allomones (of adaptive value to the organism emitting them) and kairomones (of adaptive value to the receiving organism, Brown et al., 1970). Subsequently, Nordlund and Lewis (1976) introduced synomone, an allelochemical where both receiver and emitter benefitted. Whittaker and Feeny (1971) stated that classification of these chemical agents was almost impossible due to their roles combining in “*almost all conceivable directions*.” They nevertheless tried to list subcategories of allomones (repellents, escape substances, suppressants, venoms, inductants, counteractants, and attractants) and kairomones (attractants, inductants, signals, and stimulants). The sheer quantity of these categories, partially overlapping in places, and with names that are not always self-explanatory, severely questions the value of such subdivisions.

In their original paper introducing the terms, Brown et al. (1970) talked about mutualistic, antagonistic, and defensive allomones, and presented examples of overlaps between pheromones, allomones, kairomones, and hormones. This gave rise to discussions about the usefulness of the terms, and whether they represented distinct chemical signals (e.g., Blum, 1974). Another term, apneumone, was defined as “*a substance emitted by a nonliving material that evokes a behavioral or physiological reaction adaptively favorable to a receiving organism, but detrimental to an organism, of another species, that may be found in or on the nonliving material”* (Nordlund and Lewis, 1976). This term has thankfully disappeared from use.

Table 1 gives an overview of the most commonly used terms used to describe chemical effects between organisms. These compounds have been studied widely in plants, bacteria and insects, and to a much lesser extent in vertebrates. We therefore set out to review interspecific odor-based effects in vertebrates (mainly in mammals, fish, and birds, but also including examples from amphibians and reptiles), with the specific aim of investigating the extent of and consistency in the use of the terms allomone, kairomone, and synomone. Based on this, we identified three problem areas, which led us to introduce a novel conceptual framework for use when studying interspecific odor-based effects in vertebrates.

**Table 1:** **Hierarchical overview of terminology commonly used to classify chemical effects within and between organisms**.

**Terminology**	**Definition**
**Infochemical**	Generic term synonymous with chemical cue (Sbarbati and Osculati, 2006).
**A. Hormone** from Greek *hormōn*, “to excite, impel, set in motion.”	Chemical messengers, which have to be carried frabom the organ where they are produced to the organ which they affect by means of the blood stream (Starling, 1905)
**B. Semiochemical** from Greek *ēmeion* “sign”	Chemicals that evoke a behavioral or physiological response in individuals of the same or other species (Sbarbati and Osculati, 2006).
**1. Pheromone** from Greek *pherein* “convey”	Substances, which are secreted to the outside by an individual and received by a second individual of the same species, in which they release a specific reaction, for example, a definite behavior or a developmental process (Karlson and Lüscher, 1959a).
**2. Allelochemical**, allelomone from Greek *allēl-* “one another”	[Semio]chemical that mediates interaction between two individuals that belong to different species (Dicke and Sabelis, 1988).
**a) Allomone** from Greek *allos* “other.”	Chemical substance produced or acquired^‡^ by an organism, which, when it contacts an individual of another species in the natural context, evokes in the receiver a behavioral or physiological reaction adaptively favorable to the emitter (Brown et al., 1970).
**b) Kairomone** from Greek *kairos* “advantage, opportunity, exploitative”	A transspecific chemical messenger the adaptive benefit of which falls on the recipient rather than on the receiver (Brown et al., 1970).
**i. Synomone** from Greek *syn, “*with or jointly”	Allelochemical, where both receiver and emitter benefit (Nordlund and Lewis, 1976), thus simultaneously an allomone and a kairomone.

‡ *Acquired refers here to odors appropriated (intact) from the food (Brown et al., 1970)*.

## What is wrong with the current view and terminology?

**Figure 1:**
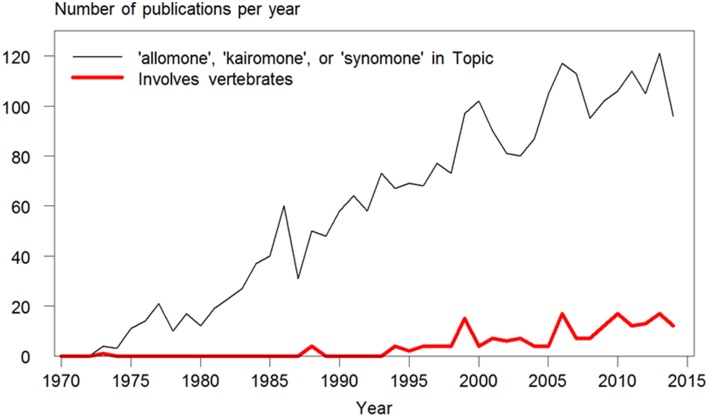
**Number of publications per year containing the terms allomone, kairomone, or synomone in their topic**. The narrow, black line shows their use overall since their coinage in the 1970's (*N* = 2635 publications) found in a search made in January 2015 in Topic (which includes words in title, keywords, and abstract) in Web of Science™ (ver. 5.16.1; Thomson Reuters © 2015). The bold, red line shows the number of publications among these, which includes vertebrate species (*N* = 184). Of these, 98 were concerned with kairomones emitted by vertebrates (mainly humans, as well as dogs, ruminants, hedgehogs, poultry, snakes, and penguins) attracting biting or stinging insects (mainly mosquitoes, as well as midges, mites, bed bugs, ticks, tsetse flies, and wasps). Only 32 publications concerning odor-based behavioral responses in vertebrate species were found when searching on any of these three terms.

The words allomone, kairomone, and synomone have been used increasingly since their coinage in the 1970's. However, among the 2644 publications found in a search on topic in Web of Science™ (ver. 5.16.1; Thomson Reuters © 2015), only 184 (7%) included vertebrates (Figure 1). Of these, 98 were concerned with kairomones emitted by vertebrates attracting biting or stinging insects (mainly humans attracting mosquitoes). Another 51 were on the subject of the use of odors by daphnia and other zooplankton to detect aquatic predators, mainly fish. The earliest use of these terms in relation to vertebrates were found in Rothschild and Ford (1973; when scientific papers could still be 50 pages long), on an odor found in newborn rabbit urine which acted as a kairomone accelerating reproduction in the rabbit flea. Overall, only 32 publications were found, which reported responses of vertebrate species to interspecific odors when searching on any of these three terms. In reality, much more research exists on this subject in vertebrates, but the three words are not used, either by omission or because the concept of who benefits does not fit the effects observed.

Ruther et al. (2002) noted that chemicals classified as kairomones had completely different biological functions for the receiving organism. Their suggestion to remedy this was to further sub-divide these compounds according to their function for the benefiting organism, thus introducing foraging, enemy-avoidance, sexual, and aggregation kairomones. However, the terms used in allelochemics are based on the assumption that we know all about the relationship between the two species considered, whereas in reality the olfactory effects are often relative, context specific, and not absolute. The designation of an odor as a kairomone or allomone is only as good as our knowledge of the relationship between the involved species.

Unlike Sbarbati and Osculati (2006), who predicted that the terms kairomone, synomone and allomone would become as popular as the terms hormone or pheromone in vertebrate studies, we are more skeptical. As we will demonstrate below, the terms—although providing a practical categorization—may constrain the way in which interspecific olfactory effects are viewed, especially in vertebrates. In the following sections, we highlight three issues relating to inter-specific odor-based effects, where the terms kairomone, synomone, and allomone do not add clarification. The three issues are innateness of the behavioral response, origin of the odor, and intent of the odorous message; these are discussed in turn below.

### Innateness of the behavioral response

Before deliberating on the innateness of vertebrate allelochemical responses, we would like to remind the reader of the same issue regarding pheromones. Although the word innate is not included in the original definition of a pheromone (Table 1), there is implicitness in the wording, where “release a specific reaction” indicates a certain automation and homogeneity of the response to a pheromone. In their original paper, Karlson and Lüscher (1959a) also invoke the principle that pheromones are effective in minute amounts, which again is more likely to be the case if the evoked response does not require any form of learning. Stowers and Marton (2005) question the notion that the response to pheromones is thought to be unalterable, and suggest that it may be context dependent. Similar caution is shown by Wyatt (2010), who describes the innate response to pheromones as conditional on development as well as context, experience, and internal state. Examples of this are perhaps more likely to be found in vertebrates (e.g., cichlids, Keller-Costa et al., 2015), which have greater cerebral development and a longer lifespan than most insects and bacteria.

Because the words used to describe interspecific odor-based effects were coined along the same root as pheromone (Karlson and Lüscher, 1959b), these “*–mones”* have the same inherent pitfalls when it comes to innateness of the responses. The definition of allomones, for example, include adaptive behavioral reactions upon contact (Table 1), thus hinting at innate responses, as was the case with pheromones. It is therefore unsurprising that it is not always clear from the literature if a given interspecific odor-based behavioral response is innate or a result of some degree of learning.

Some allelochemical responses in vertebrates appear to be innate. Snakes have been found to use chemical cues from prey species when choosing a site to wait for passing prey to ambush, even without prior experience with the prey (Clark, 2004). A good example of an un-conditioned olfactory response in vertebrates is the startle or freezing behavior of prey animals when exposed to the odor of a predator (Apfelbach et al., 2005). Kangaroos persistently avoid an area of highly palatable food containing predator-related odor cues (dingo urine or feces), and no habituation occur even in the absence of a predator (Parsons and Blumstein, 2010). Papes et al. (2010) observed increased ACTH levels and innate avoidance and risk assessment behaviors in mice exposed to odors obtained from natural mice predators [cat (neck swab), snake (shed skin), and rat (urine)], but not when exposed to rabbit urine. Black-tailed deer from a population with no exposure to wolves for a century showed reduced feeding and increased sniffing when confronted with wolf urine, as compared with black bear urine, gasoline, Cologne, and water at a feeding station (Chamaillé-Jammes et al., 2014). One of the most widely known and studied predator odors is TMT (trimethylthiazoline), which was identified in fox feces almost 40 years ago (Vernet-Maury et al., 1977; Vernet-Maury, 1980). Although some authors have expressed doubt about the kairomonal properties of TMT (McGregor et al., 2002; Fortes-Marco et al., 2013), Fendt and Endres (2008) found clear fear-evoking effects of this molecule. Indeed, mice cannot be conditioned to associate TMT with a (positive) reward as the innate avoidance response is too strong (Kobayakawa et al., 2007). Recently, another molecule (2-phenylethylamine) has been shown to trigger hard-wired aversion circuits in the rodent brain and to provoke danger-associated behavioral responses in mice and rats (Ferrero et al., 2011).

However, it is sometimes difficult to ascertain if an observed olfactory response is truly innate. Cichlids exposed to conspecific alarm cues paired with predator odor while embryos (*in ovo*) showed antipredator behavior post-hatch when exposed to predator odor alone (Nelson et al., 2013). This conditioned response happened at an early stage of development, and could easily be confused with an innate reaction to the smell of predators.

Voluntary behavioral responses, that are not innate, are learned. Animals, often dogs, can be trained to detect certain odorants. These compounds can be of non-biological origin, such as explosives and certain drugs, but sometimes the odors are found in nature, and may even arise from different species. However, it is important to distinguish between human-instigated odor-based learning and allelochemical effects. Rats and dogs have been trained to detect estrus odors in cattle (Kiddy et al., 1978; Ladewig and Hart, 1981; Hawk et al., 1984), and to detect the smell of diseases, cadavers, and even bumble-bee nests (e.g., Waters et al., 2011; Bijland et al., 2013). These are not allelochemical effects. Nevertheless, many allelochemical effects *are* a direct result of the animal learning to associate a given odor with the likelihood of an event, such as the presence of a predator or a potential sexual encounter. In addition, as was argued with pheromones, seemingly innate responses may improve with training, such as the sexual response of male rats to estrus odors (Nielsen et al., 2013).

Many examples can be found of learned interspecific odor-based responses in vertebrates. Gazdewich and Chivers (2002) found that minnows learned to recognize the odor of a predator fish when it was coupled with a minnow alarm cue, and that this conditioned response improved minnow survival. Based on chemical cues some lizard species are able to discriminate among similar predators that pose different levels of threat (Lloyd et al., 2009). Even humans, whose olfactory capacities are more modest, are able to recognize the smell of their own dog without indicating bias in terms of odor strength or pleasantness (Wells and Hepper, 2000); an ability the authors describe as “*acquired without conscious effort.”*

In conclusion, the use of terms like allomone and kairomone does not make it clear whether a reported allelochemical response is innate or not. Instead, it adds a layer of confusion, as some use the terms to imply innateness, whereas others apply the terms more broadly. From the examples given here it is apparent that although some interspecific odor-based behavioral responses are innate, they may still change with experience. An odor which is initially neutral to a receiver can become an allomone or kairomone through associative learning. Also, a kairomone can become an allomone, as shown in the intriguing example of Toxoplasmosis-infected rats, where the response to cat odors change from antipredator avoidance behavior to attraction and sexual behavior through altered neural activity in limbic brain areas caused by the parasite (House et al., 2011).

### Origin of the odor

In most uses of allelochemic terminology, the odor in question is produced by one of the species involved—often for the specific purpose of allelochemics. In one of the original descriptions of interspecific chemical messengers, the notion that an allomone can be acquired by the emitter refers to the situation where the odor is appropriated (intact) from the food (Brown et al., 1970), and thus is still originating from within the emitter, albeit not produced *de novo*. An example of this is given by Cox et al. (2010), who found that goats and kangaroos showed stronger aversion when they were exposed to the odor from a tiger fed goat or kangaroo, respectively, compared with all other predator–diet combinations. Similarly, salmon display antipredator behavior when exposed to water scented with cues from an otter, but only if the otter has been fed on a salmon diet (Roberts and de Leaniz, 2011). Wyatt (2010) raises a similar issue with pheromones, which he also suggests could be collected from the surroundings, and show individual variations in compound proportions and odor strength.

The role of the microbiota in the production of animal odors also needs to be mentioned. Microbial by-products may contribute a large part of odorants emitted by the animal. Wyatt (2010) distinguishes between pheromones and signature mixture, the latter being an individual's distinctive mix of odorous molecules (odorants), which conspecifics can learn and use for individual recognition. For example, the Indian mongoose uses anal pocket bacteria metabolites as an odorant signature (Ezenwa and Williams, 2014), and we would assign this smell as originating from the individual mongoose. In the case of microbiota by-products arising from an animal cadaver, the determination of the origin of the odor may be trickier. This is the case in zebrafish displaying a pronounced innate behavioral aversion to cadaverine and putrescine, two diamines emanating from decaying flesh (Hussain et al., 2013). Do those diamines still belong to the semiochemical category as originating from a—now dead—animal? On this basis, Dicke and Sabelis suggested already in 1988 to eliminate the origin criterion from the terminology, and to use the cost-benefit criterion as the sole determinant of infochemical subdivisions.

The message received from an odor emanating from an animal may differ dependent on where the odor is produced. Skin and fur-derived predator odors appear more efficient in evoking fear responses in prey than those derived from urine or feces (Apfelbach et al., 2005; Fendt and Endres, 2008), perhaps because the latter indicates “*a predator was here”* whereas the former could mean “*a predator is here.”* Odors derived from cat collars and cat fur can induce long lasting (up to 4 days) effects in rats following a single exposure (May et al., 2012). Incidentally, male mice can exhibit aggression in response to their own urine when it is presented on the body of a castrated mouse (Stowers and Marton, 2005). This also illustrates how the perception of an odor is highly influenced by the context, as male mice do not usually react aggressively to their own urine patches.

Mammals have also been found to anoint themselves with odors of external origin. This is commonly seen when dogs roll themselves in pungent substances, but the function of this self-scenting is debated. Hyenas show a preference for rolling in animal-based odors, and receive more attention from conspecifics when smelling of carrion (Drea et al., 2002). Ryon et al. (1986) found that wolves preferred to rub themselves in strong-smelling, manufactured odors (perfume and motor oil) above carnivore odors, and that sheep and horse feces did not elicit rubbing in wolves. Among the causal explanations for these observed behaviors is status advertisement, where dominant animals seek to stand out by adding complexity to and increasing the range of their odorous presence (Gosling and McKay, 1990). Some squirrel species anoint themselves with the scent of snakes, by chewing shed snake-skin and subsequently lick their own fur (Clucas et al., 2008). The authors suggest that this is an anti-predatory behavior, and fossil data suggest that such predator scent application in squirrels is ancient in origin (Clucas et al., 2010). Please note that this example would not be included as an allomone in the original definition of the term, as the scent did not originate from within the emitter.

Sometimes the same chemical compounds are found to act as semiochemicals for more than one species, the most famous example being a female sex pheromone shared by the Asian elephant and several species of moth (Rasmussen et al., 1996). The use of the same molecules by different species and even phyla may reflect chemical constraints relating to stability, volatility, or toxicity (Wyatt, 2010). Predator odors can also be hijacked by prey on an evolutionary scale (Papes et al., 2010), and a mouse alarm cue (2-*sec*-butyl-4,5-dihydrothiazole; Novotny et al., 1985) has been found to be similar in structure to sulfur-containing scents from certain predators, such as stoat, fox, and bobcat (Brechbühl et al., 2013). Some animals, such as certain fish species, are able to generalize predator odor recognition across predators within the same family (Ferrari et al., 2007). Rodents are able to detect estrus odors without prior training in a number of non-rodent species (horse and fox: Rampin et al., 2006; cattle: Rameshkumar et al., 2008), indicating a commonality in the estrus odor bouquet of mammals. A molecule (sulcatone) found to be associated with estrus in mammals (Nielsen et al., 2013) is also a human-derived mosquito repellent (Logan et al., 2009, 2010). Thus, the same odor may originate from different sources, some of which are of no biological relevance to the receiver of the odor.

In conclusion, the origins of odors used in a semiochemical context are quite diverse. They may be synthesized by the emitter, but may also arise from microbial by-products, from food items, or from the surroundings. Odors derived from different body areas of an animal may carry different meanings to the receiver, the same odorant can originate from different species, and an odor may be perceived to be of a different origin than is the case. We therefore agree with Dicke and Sabelis (1988) that an odor's origin should not influence whether it can be defined as an allelochemical.

### Intent of the odorous message

The use of terms like signal, messenger, and communication in the descriptions of allelochemical functions gives an inherent meaning of intent to the odor involved in the interaction. In evolutionary terms, a signal (or sign-stimuli) implies that the function of the stimuli—in this case an emitted odor—has been favored during natural selection to evoke specific behavioral responses in the receiving organism. Indeed, Whittaker and Feeny (1971) saw the evolution of allelochemic agents as a balance between metabolic cost and natural selection. In the definition of allelochemicals, the effect achieved by the emitter (for allomones) and receiver (for kairomones) has to be adaptive (Table 1; Dicke and Sabelis, 1988), in order for these effects to have been sustained through the evolutionary development of the species in question. In the case of allomones, this could indicate that an animal emitting an allomone to attract or deter another species do so for this purpose. However, this would imply not only that the odor is being actively released, but also synthesized by the emitter, which we questioned in the previous section. It seems quite unlikely according to the current view of evolutionary processes. For example, specific peptides secreted by a West-African frog species allow it to live in the underground nests of certain ants that otherwise attack and sting intruders (Roedel et al., 2013). These frogs do not secrete such specific substances as a result of contact with the ants or due to particular food items; the skin secretion is not deliberate, but simply the consequence of a serendipitous mutation allowing this frog species to live in a dry environment and protected from intruders by the ants (Roedel et al., 2013). Thus, a more appropriate way to view signaling is proposed by Scarantino (2010), who talks instead about natural information, i.e., information that is grounded in reliable correlations between types of events, such as smoke signaling fire. He encourages us to think of animal signals in a similar manner as “*carriers of natural information,”* as opposed to information being encoded in animal signals.

Viewing interspecific odors in this way also makes it less important from where the odor originates. The snake-odor anointed squirrels mentioned earlier are simply using an odor which carries natural information (“*I am a snake*”) to squirrel predators. This may also explain commonalities across species in odor preferences. Mandairon et al. (2009) found that odors rated pleasant by humans were also attractive to mice, suggesting that odor preference may be partially predetermined, based on the physicochemical structure of the odorants (Khan et al., 2007; Secundo et al., 2014). Similarities in odors serving the same purpose could also be included, such as the finding that the odor of male goats (bucks) can be used in the same way as that of male sheep (rams) to promote reproductive receptiveness of ewes (Rosa and Bryant, 2002). In stressed mice, the appeasing effects arising from the smell of conspecifics can be achieved also by exposure to odors from species that are evolutionary close (the Rodentia subclass; Cherng et al., 2012).

Odors as carriers of natural information also make it easier to reconcile divergent messages of the same odor into the concept of allelochemics. The smell of trimethylamine, an odor associated by humans with bad breath and spoiled food, is aversive to rats but attractive to mice (Li et al., 2013). As mouse urine contains high concentrations of trimethylamine, the authors conclude that this may be part of aversive allomones released by mice for defensive behavior against predators, such as rats. On the other hand, not all interspecific odor-based effects can be explained accordingly. The increased play-like behavior seen in cats when exposed to the smell of catnip (Ellis and Wells, 2010) remains enigmatic. The response is genetically determined, but may not develop fully until 3 months of age, and catnip often produces a distinct avoidance response in young kittens (Todd, 1962).

In conclusion, by viewing odors giving rise to interspecific effects as carriers of natural information (Scarantino, 2010), it allows us to include a broader spectrum of olfactory effects in allelochemics. This does not preclude the adaptiveness of the odor-based response, but moves the emphasis from the synthesis of the odor to the information it contains.

## A novel conceptual framework for the study of interspecific odor-based effects

Given the criticisms raised above concerning the usefulness of the terms allomone, kairomone, and synomone when studying vertebrates, could we find another, less constraining way to look at behavioral responses to interspecific odors? Instead of replacing the terms used in allelochemic interactions, we suggest to take a step back and view the concept from a different perspective. Below we propose a simple, yet novel conceptual framework for use when studying interspecific odor-based effects in vertebrates. It is based on two parameters, which are already widely used and referred to in the literature concerning allelochemics: one being the valence of the odor to the recipient and the other the learning involved for the odor-based response to occur.

### Odor valence and learning

Valence is a useful way to structure the behavioral response to odors (Root et al., 2014), as fast processing of an odor's meaning is important for survival and reproduction (Knaden and Hansson, 2014). Odors with a positive valence are attractive to the recipient, whereas odors with a negative valence are aversive; we thus assign valence to an odor based on the behavioral response observed when exposed to said odor. It is important to bear in mind that valence is not necessarily a fixed quality of the odor, but the motivational significance that a given odor carries to a recipient animal. This may vary between individuals of the same species dependent on sex, physiological state, previous experience, and degree of learning. Nevertheless, when using current animal models, existing behavioral tests can be used to measure relative valence. Understanding the development of aversion and attraction to odors and the mechanisms behind divergent olfactory responses has been highlighted as a model for deciphering sensory systems (Li and Liberles, 2015). Also, Secundo et al. (2014) found the primary dimension of olfactory perception in humans relates to odorant pleasantness on an axis ranging from very unpleasant to very pleasant. Although positive and negative valences are not processed by specific olfactory subsystems in mice, the dorsal olfactory bulb appear to govern avoidance behavior (Kobayakawa et al., 2007; Knaden and Hansson, 2014). A specific valence may still give rise to a specific neuronal pattern, such as the split representation of attractive and repellent odorants found in the antennal lobe of Drosophila (Ai et al., 2010; Knaden et al., 2012; Min et al., 2013). This has also been found in the olfactory tubercle of mice (Gadziola et al., 2015) and rats (Rampin et al., 2012). In the ventral striatum of the rodent brain, encoding of an odor is not fixed, but depends on odor valence; i.e., neurons in this brain region acquire a selective activation to odor exposure through associative learning, and the valence can be reversed for the same odor by the same method (Setlow et al., 2003; Gadziola et al., 2015). In addition, the amygdala has been found to play an essential part in the encoding of stimuli with affective value (Schoenbaum et al., 1999; Armony, 2013; Janak and Tye, 2015). In rodents some amygdala neurons respond to the odor of a predator (Dielenberg and McGregor, 2001; Govic and Paolini, 2015; Pérez-Gómez et al., 2015); and lesioning or inactivation of some amygdala subnuclei can eradicate the fear inducing effect of predator odor (reviewed in Takahashi, 2014). Laterality between stimuli of different valence, with positive stimuli encoded in the left and negative in the right amygdala has also been demonstrated (Young and Williams, 2010, 2013).

As discussed earlier, it is not always clear if an odor-based response is innate when allelochemics are discussed in the literature. It is, however, important for the interpretation of odor-based responses to know the extent to which it is dependent on prior experience and learning, and to take into account the plasticity of seemingly innate responses with time. This has also been highlighted by the search for specific brain regions associated with these two types of responses. For aversive odors, Kobayakawa et al. (2007) found separate sets of glomeruli in the olfactory bulb being dedicated to innate and learned responses, respectively. As was the case for valence, the amygdala is also important for both innate and learned behavioral responses to odors. The posterolateral, medial and cortical amygdala play a critical role in innate odor-driven behaviors (Martinez et al., 2011; Sosulski et al., 2011; Root et al., 2014). This was illustratively shown in the work by Blanchard and Blanchard (1972), as one of their rats with amygdaloid damage “*climbed onto the [sedated] cat's back and head, and began to nibble on the cat's ear…the cat seized and briefly shook the rat… After the cat released this rat, the rat climbed back onto the cat.”* In contrast, the basolateral amygdala appears to be involved in learned, but not innate fear responses (Ribeiro et al., 2011; Sparta et al., 2014). Also, epigenetic changes in the medial amygdala are behind the change in valence of cat odors mentioned earlier for Toxoplamosis infected rats (Hari Dass and Vyas, 2014). This may be similar to the finding that activation of the lateral amygdala caused by conditioned fear can be suppressed by external events, such as the social buffering caused by the presence of conspecifics (Kiyokawa et al., 2012; Fuzzo et al., 2015).

In conclusion, innateness of the behavioral response to certain odors as well as the learning associated with odor experience are linked to odor valence, both at the processing level and in terms of how an odor obtains a given valence. They are thus important factors involved in many odor-based responses, and therefore lend themselves as obvious candidates for constructing an alternative or complementary conceptual framework to the existing view of allelochemics with which to model interspecific odor-based effects.

### The model

**Figure 2:**
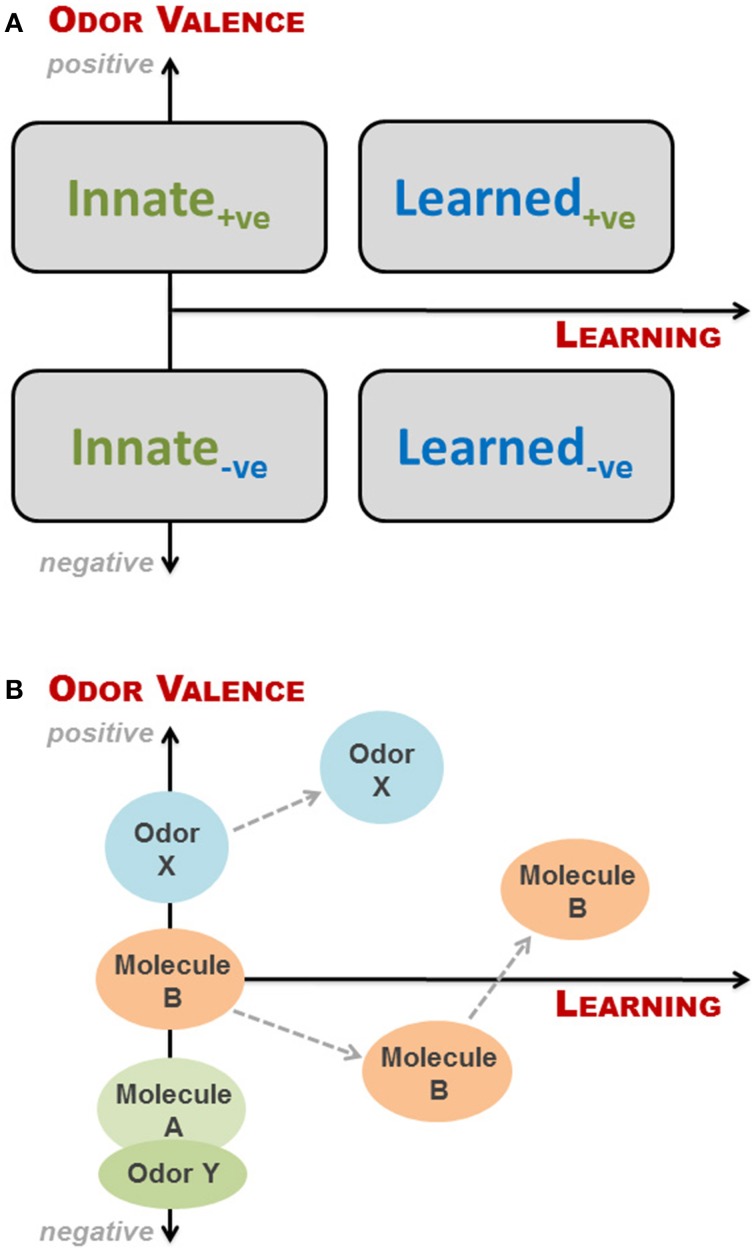
**Conceptual framework to describe interspecific olfactory effects for a given animal at a given time**. **(A)** The simplest categorization of odor-based behavioral responses is a binomial split into either innate or learned responses. Likewise, odor valence can be positive (+ve; attractive odors) or negative (−ve; aversive odors). These combine into four groupings, as depicted here by rectangles. However, odors can be more or less aversive, and odor-based behavioral responses may change with learning, even when the initial response is innate. We have therefore superimposed the four groupings onto a continual representation of learning and valence, expressing *odor valence* (y-axis) as a function of *learning* (x-axis), where positive and negative parts of the y-axis indicate degree of attraction and aversion, respectively. It is important to note that odors, which do not elicit an innate behavioral response, have no valence, i.e., are neutral to the animal and thus placed at the origin; Innate responses require no learning, but may be hard to determine and to differentiate from those arising from odors experienced in *utero* via amniotic fluid; **(B)** Examples of how different odors may be categorized relative to each other within the conceptual framework, and how odor valence may change with learning. Odor Y could be the natural smell of a predator, whereas molecule A is TMT or 2-phenylethylamine. Both evoke innate responses, but the natural odor is more fear-inducing. Molecule B is an initially neutral odor, which the animal first learns to find aversive, and subsequently associate with a positive experience. Finally, odor X may be the smell of estrus to a male before and after he gains sexual experience.

The simplest way of looking at valence and learning is to view them as two binomial parameters: if an odor has a valence (to a recipient) it is either positive (attractive) or negative (aversive), and a given odor response can be either innate or learned. These combine into four groupings, as illustrated in Figure 2A. This is, however, a fairly rough division as odors can be more or less aversive, and odor-based behavioral responses may change as a result of learning, even when the initial response is innate, as discussed above. We have therefore, in Figure 2A, superimposed the four groupings onto a continual representation of learning and valence, expressing *odor valence* (y-axis) as a function of *learning* (x-axis), where positive and negative parts of the y-axis indicate degree of attraction and aversion, respectively. As described in the previous section, the valence of an odor to an animal is determined by studying the behavioral response of the animal when exposed to said odor. If aversion or attraction is observed at first exposure to the odor, the behavioral response is innate. If no behavioral response is seen upon first exposure, the valence of the odor cannot be determined or is zero (neutral odor). In principle, for a given animal we can place any given odor along the y-axis dependent on the response of the animal at first exposure. However, as soon as the animal has experienced an odor, some degree of learning is taking place, which may or may not change the odor's valence. Thus, valence is a function of learning, where innateness/no learning is at *x* = 0 (Figure 2A); in other words odors, which do not elicit an innate behavioral response, have no valence, i.e., are neutral to the animal and thus placed at the origin. It is important to emphasize that innateness refer to the behavioral response, and not the valence (which is a consequence of the former).

The proposed conceptual framework thus consists of the two axes representing odor valence and learning, without necessarily ranking different odors *a priori* or specifying the type of learning involved. It is important to bear in mind that this is just a model, which is a way to simplify reality, in the same way that two Lego® bricks can represent an airplane. Although we may place odors associated with sex or food as being positive, and predators as negative, the relative ranking of odors is highly reliant on context. During rutting season, male ruminants may go for days without eating in search of a female in estrus (Whittle et al., 2000), and her smell is thus likely to be more attractive than food at this point in time; as soon as the breeding season has ended, the relative ranking of food odors will rise again. In a similar way, the smell of a conspecific may be associated with family or an intruder, respectively, depending on the situation. Novel food odors may be attractive to omnivorous species, such as pigs, but initially aversive to more neophobic animals, such as rats. Also, when dealing with complex odors of natural origin, different components of the smell may give rise to quite different responses, as seen when male rats were exposed to feces from vixens in estrus: Rampin et al. (2006) found the behavior of the rats shifting between freezing and penile erections, thus responding to the scent of a predator and a female in estrus, respectively.

Innate behavioral responses require no learning, but they may be hard to determine and to differentiate from those arising from odors experienced in *utero* via amniotic fluid. An example of this, although not interspecific, is the suckling response of newborn mice when presented with a nipple for the first time. Logan et al. (2012) elegantly showed that this response, which appeared innate, was elicited by the presence of signature odors—found in the amniotic fluid—that are learned and recognized prior to first suckling. Learned responses based on imprinting have only a short time window in which to be acquired; these are thus more likely to be intraspecific. The association of odors with positive or negative events has been amply illustrated in the literature, either via classical (e.g., Kvitvik et al., 2010) or operant conditioning (e.g., Rokni et al., 2014), whereas more complex learning paradigms include discrimination of several odors or odorant mixtures, as well as latent and insight learning. In Figure 2B are shown examples of how the valence of an odor may change with learning and how one odor may be categorized relative to another within the conceptual framework.

The strength of this model is in its simplicity, yet it still allows us to display the complexity of dynamic odor relationships. It is not meant to replace the terms allomone, kairomone, and synomone, but to offer an alternative viewpoint from which to investigate issues relating to allelochemics, just as the ABO and Rh blood group systems describe different aspects of an individual's blood type. A model can be used not only as a proxy to unravel complex interactions, but also as a media for organizing knowledge integration, as well as a playground for testing assumptions (Martin, 2015). We can envisage the model being expanded to contain additional variables, such as time, odor complexity or odor concentration, depending on the testing paradigm. Indeed, the concepts of who benefits from the interaction could be introduced along the z-axis. The model may be used to construct a diagram of different parts of the brain involved in innate and learned odor-based responses for odors of positive and negative valence, respectively. Finally, it need not be constrained to interspecific odor-based effects, but may help visualize the learning involved in the sculpting of some innate pheromonal responses (Stowers and Marton, 2005; Wyatt, 2010), as well as other odor-based responses.

## Conclusion

We have demonstrated the constraining properties and the limits to the usefulness of the terms allomone, kairomone, and synomone when studying interspecific odor-based effects in vertebrates. We do not propose to replace the terms used in allelochemic interactions, but instead to view the concept from a different perspective. We present a simple, yet novel conceptual framework based on two parameters: valence of the odor to the recipient and the learning involved in the observed behavioral response to the odor. This model provides a unifying framework for use when studying interspecific odor-based effects, particularly in vertebrates.

### Conflict of interest statement

The authors declare that the research was conducted in the absence of any commercial or financial relationships that could be construed as a potential conflict of interest.
